# Characterization of the complete chloroplast genome of *Cycas ferruginea*, a vulnerable species

**DOI:** 10.1080/23802359.2022.2082894

**Published:** 2022-06-14

**Authors:** Xuan Yang, Tao Deng, Wenxiu Tang, Tingting Wu

**Affiliations:** a College of Tourism and Landscape Architecture, Guilin University of Technology, Guilin, China; b Guangxi Institute of Botany, Guangxi Zhuang Autonomous Region and Chinese Academy of Sciences, Guilin, China

**Keywords:** *Cycas ferruginea*, complete chloroplast genome, phylogenetic analysis

## Abstract

*Cycas ferruginea* F. N. Wei (1994) is recorded in the list of wild plant protection in China as a national first-class protected plant. The complete chloroplast genome of *C. ferruginea* was analyzed for the first time in this article. The genome is 162,045 bp in length, which contains a pair of inverted repeats (IRs) of 25,048 bp each, a large single-copy (LSC) region of 88,827 bp, and a small single-copy (SSC) region of 23,122 bp. The genome comprises a total of 130 encoded genes, including 85 protein-coding genes, eight ribosomal RNA genes, and 37 transfer RNA genes. The total GC content is 39.44%, and the corresponding values of the LSC, SSC, and IRs are 38.73%, 36.56%, and 42.02%, respectively. The phylogenetic relationships were reconstructed based on the complete chloroplast genome sequences of 16 species. Results showed that *C. ferruginea* is close to *C. debaoensis*, *C. bifida*, and *C. szechuanensis*.

*Cycas* is often considered a living fossil, thereby providing a unique model for revealing the evolution of spermatophytes (Zhong et al. 2011). *Cycas ferruginea* F. N. Wei (1994), a member of cycad family (Cycadaceae, *Cycas*) (Wei 1994), is recorded in the list of wild plant protection in China as a national first-class protected plant. It grows on semi-shaded rocky crevices in broad-leaved forests in limestone mountains at altitudes of 200–500 m. The leaves and petiole of *C. ferruginea* are densely rusty brown tomentose when young. Based on the assessment of the latest IUCN red list (http://www.iucnredlist.org/search), this species is considered to be Near Threatened. Its geographical range is limited to China and Vietnam.

For this study, the fresh leaves of a single *C. ferruginea* were collected from Guangxi Institute of Botany, Guilin, China (110°17′57.57″ E, 25°4′58.67″ N). *C. ferruginea* sampling was permitted by the Ministry of Science and Technology of the People’s Republic of China (project number 2017 FY100100). The collection and handling of *C. ferruginea* followed the guidelines of the International Union for Conservation of Nature (IUCN) Policy Research involving species at risk of extinction, the Convention on Biological Diversity and the Convention on the Trade in Endangered Species of Wild Fauna and Flora. A specimen was deposited at Herbarium of Guangxi Institute of Botany (http://www.gxib.cn/spIBK/, Z. C. Lu, email: zhaocenlu@163.com) under voucher number IBK00438289. Total genomic DNA was extracted by following the cetyltrimethyl ammonium bromide (CTAB) protocol (Doyle and Doyle 1987). A total of 4.3 G raw data from Illumina Hiseq Platform were screened and assembled into a complete chloroplast genome by GetOrganelle in a typical way (Jin et al. 2020). The assembled complete chloroplast genome sequences were annotated by using the tool CPGAVAS2, with *Cycas bifida* (MW_900434) as reference (Shi et al. 2019; Zhang et al. 2021).

Study results show that the assembled plastid genome of *C. ferruginea* is 162,045 bp in length (GenBank accession MZ977201), which contains a pair of inverted repeats (IRs) of 25,048 bp each, a large single-copy (LSC) region of 88,827 bp, and a small single-copy (SSC) region of 23,122 bp. The genome comprises a total of 130 encoded genes, including 85 protein-coding genes, eight ribosomal RNA genes, and 37 transfer RNA genes. The total GC content is 39.44%, and the corresponding values of the LSC, SSC, and IRs are 38.73%, 36.56%, and 42.02%, respectively.

The phylogenetic tree was constructed using the complete chloroplast genomes of 15 published species within Cycadales and one outgroup species. All the 16 sequences downloaded from NCBI were aligned by using MUSCLE (Edgar 2004). The maximum-likelihood (ML) tree with 1000 bootstrap replicates was performed with RaxMl v 8.2.12 (Stamatakis 2014). Cycadaceae and Zamiaceae constitute two clades of Cycadales (Figure 1). The species of Cycadaceae are distributed in Asia (China, Vietnam, and Japan), while those of Zamiaceae are distributed in North America (Mexico), Oceania (Australia), and Africa (South Africa). Furthermore, *C. ferruginea* is close to *C. debaoensis*, *C. bifida*, and *C. szechuanensis*. The complete chloroplast of *C. ferruginea* contributes to the growing number of chloroplast genomes for phylogenetic and evolutionary studies in Cycads.

**Figure 1.**
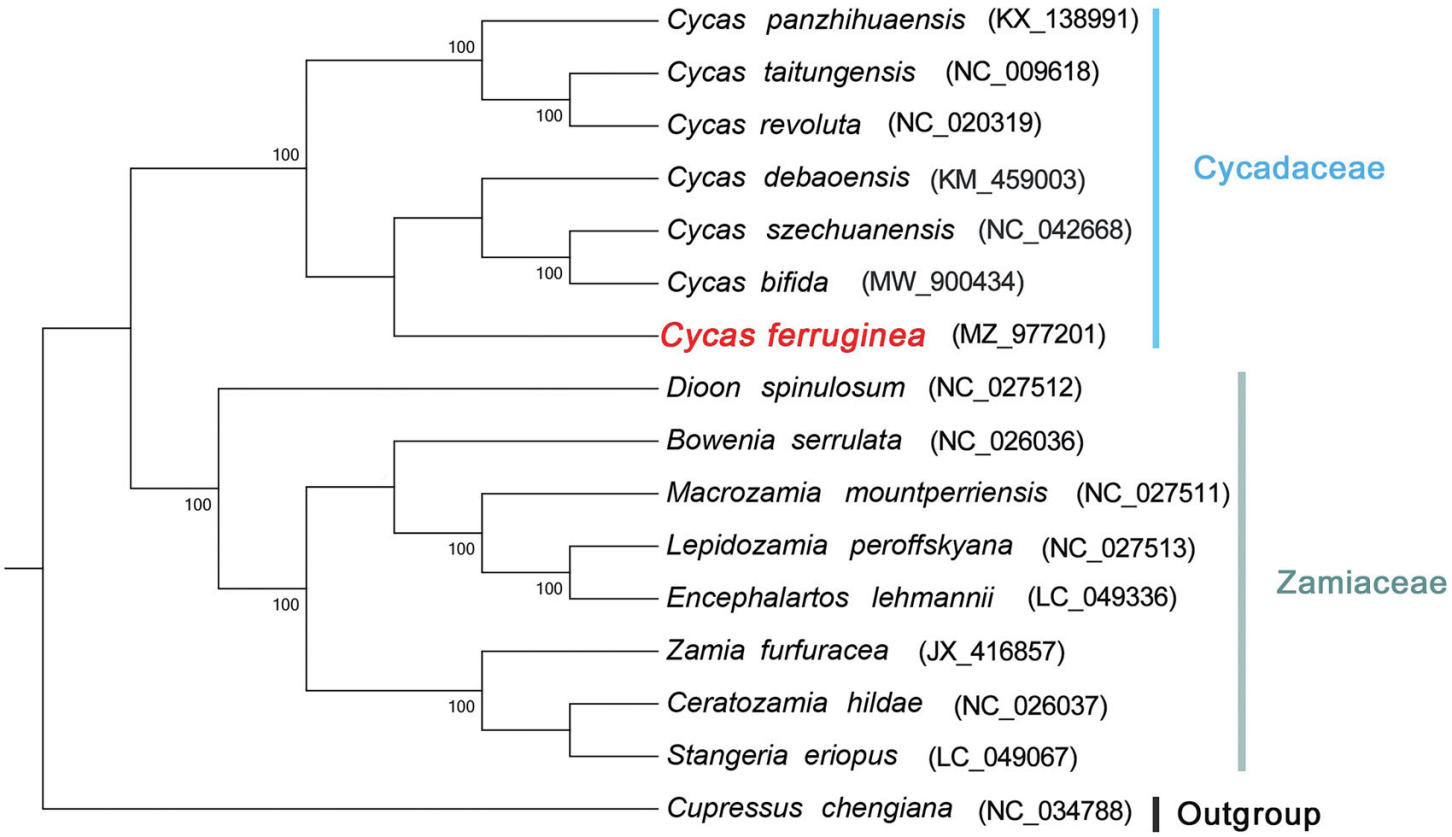
Maximum-likelihood tree based on the chloroplast gene sequences of *C. ferruginea* and 15 other species. The bootstrap values are shown on the nodes, and the species and GenBank accession number are shown at the end of each branch.

## Author contributions

Wenxiu Tang: contributed to the conception of the study; Tao Deng: contributed significantly to analysis and manuscript preparation; Xuan Yang: performed the experiment and the data analysis, wrote the manuscript; Tingting Wu: helped perform the analysis with constructive discussions. We agree to be accountable for all aspects of the work.

## Data Availability

This data that support the findings of this study are openly available in GenBank of NCBI at https://www.ncbi.nlm.nih.gov, under the accession no. MZ977201. The associated Bio-Project, SRA, and Bio-Sample numbers are PRJNA780108, SRR16938431, and SAMN23102654, respectively. Treefile of 16 species and genes for phylogenetic analysis were deposited at Figshare: https://doi.org/10.6084/m9.figshare.17008474.v1. The data are morally correct and does not violate the protection of human subjects or other valid ethical, privacy, or security concerns.
